# The Role of the bHLH Protein Hairy in Morphogenetic Furrow Progression in the Developing *Drosophila* Eye

**DOI:** 10.1371/journal.pone.0047503

**Published:** 2012-10-31

**Authors:** Abhishek Bhattacharya, Nicholas E. Baker

**Affiliations:** 1 Department of Genetics, Albert Einstein College of Medicine, Bronx, New York, United States of America; 2 Department of Developmental and Molecular Biology, Albert Einstein College of Medicine, Bronx, New York, United States of America; 3 Department of Ophthalmology and Visual Sciences, Albert Einstein College of Medicine, Bronx, New York, United States of America; VIB and KU Leuven, Belgium

## Abstract

In *Drosophila* eye development, a wave of differentiation follows a morphogenetic furrow progressing across the eye imaginal disc. This is subject to negative regulation attributed to the HLH repressor proteins Hairy and Extramacrochaete. Recent studies identify negative feedback on the bHLH gene *daughterless* as one of the main functions of *extramacrochaete*. Here the role of *hairy* was assessed in relation to *daughterless* and other HLH genes. Hairy was not found to regulate the expression of Daughterless, Extramacrochaete or Atonal, and Hairy expression was largely unregulated by these other genes. Null alleles of *hairy* did not alter the rate or pattern of differentiation, either alone or in the absence of Extramacrochaete. These findings question whether *hairy* is an important regulator of the progression of retinal differentiation in *Drosophila*, alone or redundantly with *extramacrochaete*.

## Introduction

Neural progenitor cells are specified within proneural regions controlled by members of the helix-loop-helix (HLH) protein family. Differentiation of ∼800 ommatidia in the *Drosophila* neural retina begins at the posterior margin of the third instar larval eye imaginal disc. The specification of the founder R8 photoreceptor precursor cells accompanies the ‘morphogenetic furrow’, a visible groove that moves anteriorly across the eye disc epithelium [1]. The mechanism by which the morphogenetic furrow advances differentiation across the eye disc has been the subject of much attention. The extracellular signaling molecules Hedgehog (Hh) and Decapentaplegic (Dpp) induce expression of the bHLH gene *atonal (ato)*, the proneural gene responsible for R8 specification, in a band of cells just anterior to the morphogenetic furrow. Notch signaling and lateral inhibition refine Ato expression from this band to the array of single R8 precursor cells that each found one ommatidium. Posterior to the morphogenetic furrow, R8 cells then recruit precursors of other photoreceptor cell types, some of which then express Hh to keep the furrow progressing. The whole retina is differentiating once the morphogenetic furrow has crossed the eye primordium, which takes about two days [2].

**Figure 1:**
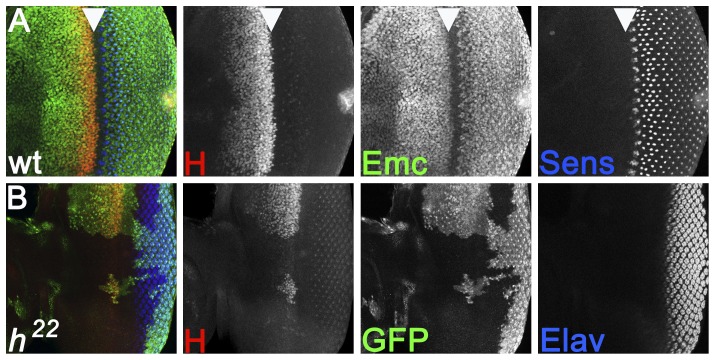
Hairy and Emc expression in the eye disc. (A) Wild type eye disc labeled for Hairy protein (red), Emc (Green) and Senseless (blue). Arrowhead indicates the downregulation of Hairy just ahead of the morphogenetic furrow. Emc is downregulated almost simultaneously; Sens reports the first steps of differentiation soon afterwards. (B) Clones of cells homozygous for the null allele *h^22^* lack almost all Hairy antigen. Eye differentiation, as recorded by the pan-neuronal marker Elav (blue), is hardly affected.

In addition to the positive regulation by Hh and Dpp, morphogenetic furrow progression is thought to be regulated negatively by two nuclear HLH proteins, Hairy and Extramacrochaetae (Figure 1A). Although clones of cells homozygous for neither *hairy* null mutations nor *emc* hypomorphic mutations affect morphogenetic furrow progression by themselves, clones of the double mutant combination result in significant faster furrow progression. This observation, along with the expression pattern of the genes, suggested that *hairy* and *emc* regulate furrow progression by redundant or overlapping mechanisms [3]. Emc is widely expressed but downregulated in the morphogenetic furrow by Hh and Dpp signaling(Figure 1A) [3], [4]. Hairy is expressed in a broad region ahead of the furrow and downregulated just anterior to the furrow by combinatorial activities of Hh and Notch signaling (Figure 1A) [5], [6], [7]. It has been proposed that the Hairy expression domain reflects cells in a ‘preproneural state’ ahead of the morphogenetic furrow, in which inhibitors such as Hairy are required to restrain proneural pathways whose activation is imminent [8].

Recently, *emc* has been described as part of a regulatory network of HLH genes [4]. According to these recent studies, effects of mutating *emc* are in fact mediated by derepressed expression of another HLH protein, Daughterless (Da) [4]. Da, the only *Drosophila* E-protein, functions as the essential heterodimer partner of Atonal in the eye [9], [10]. In addition to regulating *da* expression, *emc*, the *Drosophila* homolog of mammalian Inhibitor of DNA-binding (Id) proteins, encodes a HLH protein without the basic DNA-binding domain and so inhibits Ato and Da activity through inactive heterodimer formation [11]. Hh and Dpp signaling therefore facilitate formation and activity of the Ato/Da heterodimer by repressing Emc expression during the time that Ato is turned on (Figure 1A) [4]. Because Emc inhibits the ability of Da expression to auto-regulate, this allows Da levels to rise in the morphogenetic furrow. Outside the morphogenetic furrow, and other proneural regions, the widespread expression of Emc sets a threshold for neurogenesis by limiting the Da expression level and proneural bHLH/Da heterodimer activity [4].

As *emc* was thought to act along with *hairy* in regulating furrow progression, these recent findings prompted us to examine how *hairy* fits into the emerging network of HLH protein cross-regulation. Because *hairy* also encodes a repressor HLH protein, *hairy* might target *da* expression, like *emc* does. Unlike Emc, Hairy is a bHLH protein that acts as a classical transcriptional repressor by sequence specific DNA binding, rather than by heterodimerization with proneural bHLH proteins [12]. Hairy is required for proper transcription of proneural genes and patterning of sensory organs in developing wing and leg, where it represses transcription of the proneural gene *achaetae* [12].

Since the original studies of hypomorphic *emc* mutations, clones of *emc* null mutant cells are now known to show faster morphogenetic furrow progression even in the presence of wild type *hairy*, and this is due to the elevated Da expression in such clones so that *emc da* double mutant clones no longer accelerate the furrow [4], [13]. The stronger phenotype of *emc* null alleles compared to *emc^1^* suggests that complete removal of *hairy* and *emc* together should have a stronger phenotype still and reveal full extent of negative regulation of differentiation by HLH proteins.

Here we explore regulatory relationships between *emc*, *hairy*, *da* and the progression of differentiation. We report that *hairy* does not seem to be regulated by or a regulator of the Da/Emc network. In addition, we find that *hairy* null alleles have no effect on morphogenetic furrow movement in the complete absence of *emc*, challenging the view that these two genes regulate morphogenetic furrow progression together. In fact, our studies of *hairy* null mutations have yet to identify any specific role for this gene in regulating the morphogenetic furrow.

## Results

### Da, Emc and Atonal expression are independent of *hairy*

**Figure 2:**
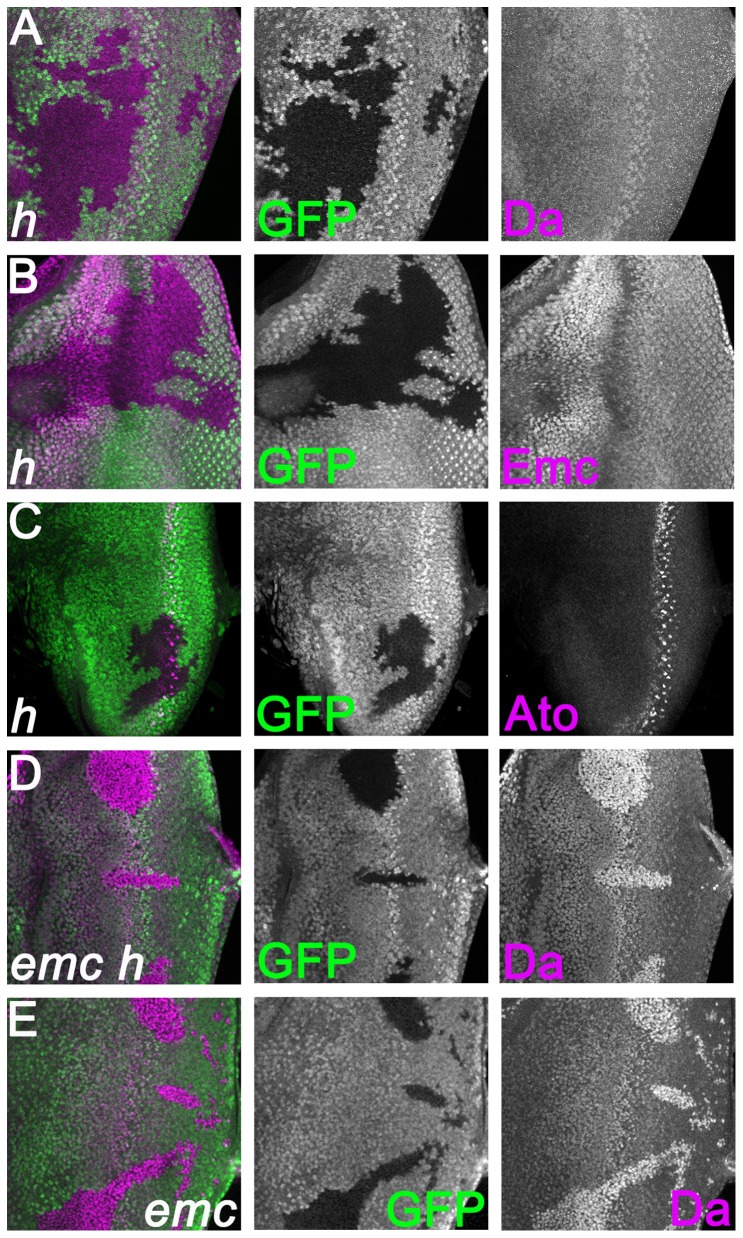
Da, Emc and Hairy expression are independent of *hairy*. Clones of homozygous mutant cells are labeled by the lack of GFP expression (green). (A) In *h^22^* mutant clones, Da expression (magenta) both inside and outside of the furrow remained unaffected. (B) In *h^22^* mutant clones, Emc expression (magenta) both inside and outside of the furrow remained unaffected. (C) In *h^22^* mutant clones, Ato expression (magenta) was unaffected. (D) Cell-autonomous high Da expression (magenta) in *emc^AP6^ h^22^* double mutant cells. Note the very similar levels of Da in *emc^AP6^ h^22^* double mutant cells and *emc^AP6^* mutant cells (compare panel E). (E) Cell-autonomous high Da expression (magenta) in *emc^AP6^* mutant cells. Note the very similar levels of Da in *emc^AP6^* mutant cells and *emc^AP6^ h^22^* double mutant cells (compare panel D). **Genotype**: (A–C) *ywhsF; h^22^* FRT80/[*Ubi-GFP*] *M*(3)67*C* FRT80; (D) *ywhsF; emc^AP6^ h^22^* FRT80/[*Ubi-GFP*] *M*(3)67*C* FRT80; (E) *ywhsF; emc^AP6^* FRT80/[*Ubi-GFP*] *M*(3)67*C* FRT80.

We investigated Da and Emc expression in the absence of *hairy* activity in clones homozygous for the null allele *h^22^*. This allele contains a stop codon within the basic region, so that a truncated protein lacking DNA-binding, dimerization, or repressor domains is predicted [5] [14]. Clones of homozygous *h^22^* cells lacked almost all Hairy antigen, with little consequence for retinal differentiation [5] (Figure 1B). Both within the morphogenetic furrow and elsewhere, Da expression remained unchanged (Figure 2A). In the case of Emc protein, expression both within the morphogenetic furrow and elsewhere also remained unchanged in *h* clones (Figure 2B). These findings suggest that *hairy* has no effect on the expression of Emc or Da. Da and Emc each form heterodimers with Ato, through which they regulate eye differentiation. We tested whether Ato was also a target of *hairy*. Ato expression remained unchanged in clones homozygous for *h^22^* (Figure 2C). Because *hairy* might act redundantly with *emc*, we examined Da expression in clones doubly null for both *emc* and *h*. Da expression was strongly elevated, as was previously seen in *emc* null clones, and the levels of Da expression appeared indistinguishable in the two genotypes (Figure 2D, E) [4].

### Emc regulates morphogenetic furrow progression independently of hairy

**Figure 3:**
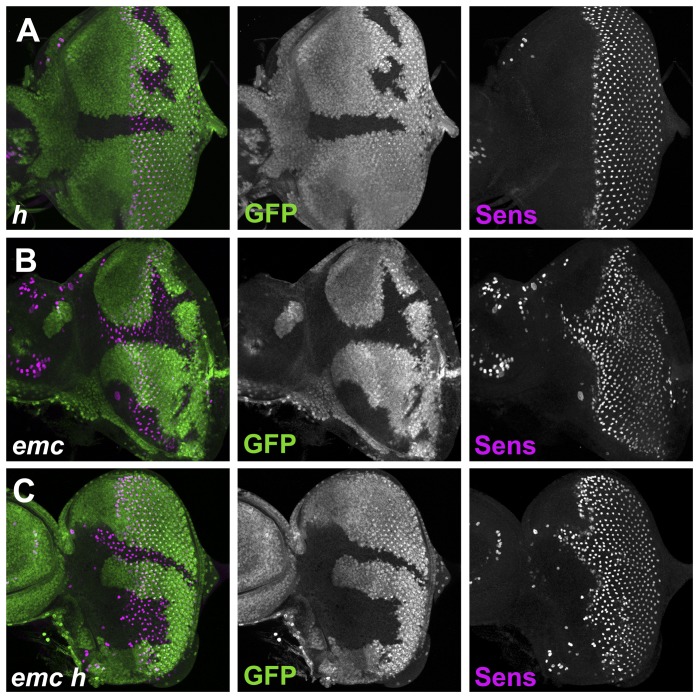
Morphogenetic furrow progression is regulated by *emc* not by *hairy*. Clones of homozygous mutant cells are labeled by the lack of GFP or LacZ expression (green). Sens labeling in magenta. (A) In *h^22^* mutant clones, differentiation remained unaffected. (B) In *emc^AP6^* mutant clones, differentiation was significantly advanced compared to neighboring tissue, more so in clones extending further in the anterior-posterior axis like the one nearer the top of the disc. (C) In *emc^AP6^ h^22^* double mutant clones, morphogenetic furrow progression was significantly advanced compared to neighboring tissue, more so in clones extending further in the anterior-posterior axis like the one nearer the top of the disc. **Genotype**: (A) *ywhsF; h^22^* FRT80/[*Ubi-GFP*] *M*(3)67*C* FRT80; (B) *ywhsF; emc^AP6^* FRT80/[*Ubi-GFP*] *M*(3)67*C* FRT80; (C) *ywhsF; emc^AP6^ h^22^* FRT80/[*Ubi-GFP*] *M*(3)67*C* FRT80.

Although clones of cells homozygous for either *hairy* null mutations or *emc* hypomorphic mutation do not affect morphogenetic furrow progression, their combination results in significantly faster furrow progression [3]. As the morphogenetic furrow also moves faster in clones of *emc* null mutations than in wild type [13], we determined whether *hairy* mutation had any further effect on morphogenetic furrow progression in the absence of *emc*. As published previously, *hairy* null mutations alone had little effect on morphogenetic furrow progression, as visualized by 22C10 antibody staining [3] (data not shown). In the present study, we also used expression of the R8 protein Senseless (Sens) as a marker for Atonal activity and morphogenetic furrow progression (Figure 1A) [15]. Sens expression was not altered in the absence of *hairy* (Figure 3A). As reported previously, the morphogenetic furrow was advanced anteriorly inside *emc* null clones [13] (Figure 3B). The morphogenetic furrow was advanced to a similar degree inside *emc h* double null clones, which lacked detectable Hairy antigen (Figure 3C and data not shown). To quantify furrow progression in *emc* and *emc h* clones, we measured the distance between the anteriormost extents of Sens expression within and outside the mutant clones. Then the distance that the furrow was advanced was compared to the extent of the clone behind the morphogenetic furrow, to estimate over what time period the difference arose. From such measurements we concluded that the onset of Sens expression progressed 1.40±0.05 times faster in *emc* null clones than in wild type tissue, and that this rate appeared independent of the size of the clone, consistent with the morphogenetic furrow moving faster through *emc* null cells at a constant rate without accelerating further. For *emc h* double mutant clones, the estimate was 1.45±0.08 times faster than wildtype. As these measures could not be distinguished statistically, there was no evidence that the morphogenetic furrow travelled faster in *emc h* clones than in *emc* null clones.

### Hairy expression is independent of *emc* and *da*

**Figure 4:**
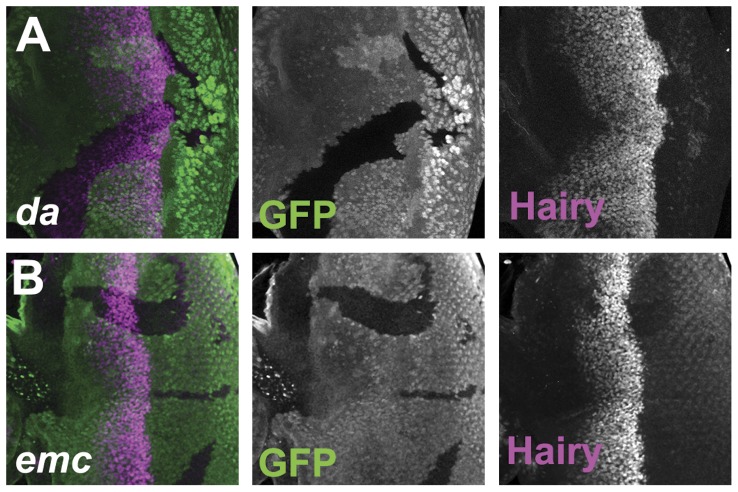
Hairy expression is independent of Emc and Da. Clones of homozygous mutant cells are labeled by the lack of GFP expression (green). (A) Hairy expression (magenta) continues longer in the center of *da* clones, indicating a requirement for *da* in non-autonomous signals that regulate Hairy, not any direct cell-autonomous effect. There may also sometimes be a non-autonomous delay in the start of Hairy expression. (B) Hairy expression (magenta) is lost slightly earlier as furrow progression occurs more rapidly in *emc* clones. **Mutant genotypes**: (A) *ywhsF; da^10^* FRT40/[*UbiGFP*] FRT40; (B) *ywhsF; emc^AP6^* FRT80/[*Ubi-GFP*] *M*(3)67*C* FRT80.

Both Emc and Da expression depend on *da* function [4]. To test whether *da* also regulates Hairy expression, clones of cells null for *da* were examined. Only minor changes in Hairy expression were observed, and as these were non-autonomous they were presumably indirect (Figure 4A). Specifically, both the onset and the termination of Hairy expression were somewhat delayed in the center of *da* null clones, but neither effect was seen close to the clone boundaries (Figure 4A). Similar results were obtained when large *da* clones were induced in a Minute background (data not shown). These data indicate that *da* is not directly required to regulate Hairy expression in the same cells, but is responsible for the expression of signals that affect Hairy expression cell-nonautonomously. Hairy downregulation in the furrow requires Notch and Hh signaling cell-autonomously [6], [7]. Therefore, lack of differentiation in the absence of *da* could affect Hairy expression because both Hh and the Notch ligand Dl are expressed by differentiated cells and depend on *ato* and *da* function [16], [17]. A similar explanation may underlie the delayed onset of Hairy expression in *da* clones, but in this case the signals that initiate Hairy expression in the anterior eye are not completely known, except that Dpp signaling contributes [7], [8].

Emc plays an important role restraining Da expression [4]. To test whether *emc* regulates Hairy expression, clones of cells null for *emc* were examined. Hairy expression also changed little in the *emc* null mutant clones (Figure 4B). Hairy downregulation in the furrow occurred slightly earlier in *emc* mutant clones, in a cell-autonomous fashion (Figure 4B). This early downregulation in *emc* clones is not surprising in light of faster morphogenetic furrow progression in the absence of *emc* [13], but an additional direct effect of *emc* on Hairy downregulation cannot be ruled out. Taken together, these findings showed that Hairy expression depends very little on either *emc* or *da*.

## Discussion

The morphogenetic furrow moves anteriorly across the eye disc under the positive influence of Hh and Dpp. The forward progression of differentiation is a consequence of the positive activation of Ato expression as well as the parallel repression of Emc, which results in elevated levels of the heterodimer partner of Ato, Da [4]. Hh and Dpp also affect the cell cycle [18], the shapes of cells in the morphogenetic furrow [19], [20], the expression of retinal determination genes [21], [22], and the sizes of nucleoli (NEB and J.Han, unpublished), although it remains to be determined whether these other processes contribute directly to neural differentiation.

This paper addresses *hairy*, a potential barrier to morphogenetic furrow movement. Hairy protein is expressed through much of the eye disc anterior to the morphogenetic furrow, and is downregulated sharply at the time that Atonal becomes active [3] (Figure 1A). Although clones of *hairy* null mutations do not affect eye differentiation, it has been thought that *hairy* acts along with *emc*. so that *emc* hypomorphs that have no effect on the morphogenetic furrow progression alone do speed up the furrow in combination with *hairy* null mutations [3]. It has been proposed that Hairy is a marker of a ‘preproneural state’, in which the presence of Hairy helps restrain incipient neurogenesis [8].

If *hairy* acts redundantly with *emc*, this might be explained by convergence on common targets, since both encode transcriptional repressors. We found, however, no noticeable effect of *hairy* null alleles on Da expression, Emc expression, or Ato expression (Figure 2). In addition, *h emc* double mutant clones appeared to have no additional effect on Da expression from that seen in *emc* clones. Since no obvious role for *hairy* in the expression of these genes was detected, the progression of the morphogenetic furrow was measured directly. Although differentiation progresses faster through cells null for *emc* than through wild type cells, removing *hairy* had no further effect on morphogenetic furrow progression. These findings provide no evidence that *hairy* acted redundantly with *emc*, since it did not regulate morphogenetic furrow progression or target gene expression when *emc* function was removed, implying that *hairy* function was not sufficient to compensate even partially for the absence of *emc*. In fact, a *hairy* null mutation has no discernible effect on the morphogenetic furrow in either the presence or absence of *emc*. There may be a small role for *emc* in regulating Hairy expression, such that Hairy is repressed slightly faster in the absence of *emc*, but even the complete absence of *hairy* has no effect on furrow progression, either in the presence or absence of *emc*. In conjunction with experiments in which Hairy did not affect morphogenetic furrow progression when over-expressed [5], these findings challenge the model that Hairy regulates morphogenetic furrow progression.

The role for *hairy* in regulating morphogenetic furrow progression was suggested because *hairy* antagonizes neurogenesis in other imaginal discs, and because *hairy* mutations enhanced the phenotype of the *emc^1^* mutant allele [3]. In addition, failure to downregulate Hairy at the morphogenetic furrow correlates with reduced differentiation in a number of mutant genotypes [7]. The neurogenic phenotype of *hairy* in other imaginal discs depends on Hairy binding to the enhancer of *achaetae* [12]. Since *achaetae* is not expressed or functional during morphogenetic furrow progression, these data offer no basis for predicting *hairy* function in the eye.

Enhancement of the *emc^1^* allele, but not the *emc^AP6^* null allele, could be explained if *hairy* contributed to *emc* function in some way, so that *hairy* function can mitigate partial loss of *emc* function by increasing the effectiveness of the remaining Emc protein, but would not affect the *emc* null phenotype. The *emc^1^*mutant allele encodes a Val-to-Glu substitution in the HLH domain, which would be expected to interfere with heterodimer formation by Emc^1^ protein, consistent with a hypomorphic phenotype [23]. We found no evidence that *hairy* contributed to the expression of Emc or to Emc function as a negative regulator of *da*. Another possibility is that Hairy protein might act through distinct mechanisms in addition to binding to specific DNA sequences. The E(spl) proteins, which contain similar domains to Hairy, can also repress gene expression when targeted to particular genes by protein-protein interactions [24]. It has not been tested whether Hairy might exhibit similar protein-protein interactions. It is also reported that the Chicken Id protein, a homolog of Emc, interacts directly with Hes1, a homolog of Hairy [25]. Thus far, however, *Drosophila* Hairy is not known to heterodimerize with Emc or any of its proneural gene targets [26], [27]. It is possible that Hairy might regulate *da* transcription in a subtle way only revealed in the *emc^1^* backgrounds. For example, Hairy repression of *da* transcription might be redundant in the presence of wild type *emc*, and not sufficient to impact *da* autoregulation in the complete absence of Emc. Detailed information concerning the thresholds of *da* transcription under different conditions would be required to assess this model.

The Hairy expression ahead of the morphogenetic furrow certainly seems to provide a marker of an early stage of eye development [8]. Consistent with this, retention of Hairy expression in mutant genotypes correlates with diminished retinal differentiation [7]. Our findings here indicate that, contrary to previous models, any contribution of Hairy to morphogenetic furrow progression is quite limited, and there is little evidence to connect it with *emc*. The possibility remains that *hairy* may function in a subtle way, perhaps redundantly with other genes, or affect processes other than furrow progression, particularly since many questions remain to be resolved concerning the transcriptional regulation of eye development, such as how *ato* expression is initiated as the furrow progresses, or all the mechanisms by which the retinal determination genes contribute to eye development [28]. It is also possible that laboratory conditions conceal the contribution of the *hairy* gene in eye development, as has been suggested for regulatory pathways that are thought to contribute temperature stability in variable environments [29].

## Materials and Methods

Primary antibodies used were monoclonal mouse anti-Daughterless [30], polyclonal rabbit anti-Extramacrochaetae [3], polyclonal guinea pig anti-Senseless [15], polyclonal guinea-pig anti-Hairy [31], monoclonal rat anti-Elav (DSHB), monoclonal mouse-anti 22C10 [32], rabbit anti-ß-Galactosidase (Cappel), mouse and rabbit anti-GFP antibodies (Invitrogen #A11120 and A11122). Secondary antibodies were Cy2- and Cy3-conjugates from Jackson Immunoresearch. Antibody was performed as described [4]. To estimate rates of furrow progression in mutant clones, the position of the differentiation wave through wild type regions of each eye disc was first determined from the Senseless expression pattern. Then the extent of differentiation both anterior and posterior to this reference was estimated from the Senseless expression pattern within the mutant clone. The number of mutant columns posterior to the limit of differentiation in wild type cells was the estimate of when the furrow began traversing mutant tissue. The ratio to the total number of mutant columns differentiating estimated the average speed of progression through the mutant clone. The measurements reported are from 7 suitably-shaped *emc* clones and 11 *emc h* clones.

## References

[1] Wolff T, Ready DF (1993) Pattern formation in the *Drosophila* retina. In: Bate M, Martinez Arias A, editors. The Development of *Drosophila melanogaster*: Cold Spring Harbor Laboratory Press. pp. 1277–1325.

[2] Roignant J-Y, Treisman JE (2009) Pattern formation in the Drosophila eye disc. Int J Dev Biol 53: 795–804. 19557685 10.1387/ijdb.072483jr PMC2713679

[3] Brown NL, Sattler SA, Paddock SW, Carroll SB (1995) *hairy* and *emc* negatively regulate morphogenetic furrow progression in the developing *Drosophila* eye. Cell 80: 879–887. 7697718 10.1016/0092-8674(95)90291-0

[4] Bhattacharya A, Baker NE (2011) A network of broadly expressed HLH genes regulates tissue-specific cell fates. Cell 147: 881–892. 22078884 10.1016/j.cell.2011.08.055 PMC3268347

[5] Brown NL, Paddock SW, Markey DR, Carroll SB (1991) hairy gene function in the Drosophila eye: normal expression is dispensable but ectopic expression alters cell fates. Development 113: 1245–1256. 1811940 10.1242/dev.113.4.1245

[6] Baonza A, Freeman M (2001) Notch signalling and the initiation of neural development in the *Drosophila* eye. Development 128: 3889–3898. 11641214 10.1242/dev.128.20.3889

[7] Fu W, Baker NE (2003) Deciphering synergistic and redundant roles of Hedgehog, Decapentaplegic and Delta that drive the wave of differentiation in Drosophila eye development. Development 130: 5229–5239. 12954721 10.1242/dev.00764

[8] Greenwood S, Struhl G (1999) Progression of the morphogenetic furrow in the *Drosophila* eye: the roles of Hedgehog, Decapentaplegic and the Raf pathway. Development 126: 5795–5808. 10572054 10.1242/dev.126.24.5795

[9] Jarman AP, Grau Y, Jan LY, Jan YN (1993) *atonal* is a proneural gene that directs chordotonal organ formation in the *Drosophila* peripheral nervous system. Cell 73: 1307–1321. 8324823 10.1016/0092-8674(93)90358-w

[10] Brown NL, Paddock SW, Sattler CA, Cronmiller C, Thomas BJ, et al (1996) *daughterless* is required for *Drosophila* photoreceptor cell determination, eye morphogenesis, and cell cycle progression. Developmental Biology 179: 65–78. 8873754 10.1006/dbio.1996.0241

[11] Campuzano S (2001) Emc, a negative HLH regulator with multiple functions in Drosophila development. Oncogene 20: 8299–8307. 11840322 10.1038/sj.onc.1205162

[12] Fisher A, Caudy M (1998) The function of hairy-related bHLH repressor proteins in cell fate decisions. Bioessays 20: 298–306. 9619101 10.1002/(SICI)1521-1878(199804)20:4<298::AID-BIES6>3.0.CO;2-M

[13] Bhattacharya A, Baker NE (2009) The HLH protein Extramacrochaetae is required for R7 cell and cone cell fates in the Drosophila eye. Dev Biol 327: 288–300. 19118542 10.1016/j.ydbio.2008.11.037 PMC2847366

[14] Wainwright SM, Ish-Horowicz D (1992) Point mutations in the Drosophila hairy gene demonstrate in vivo requirements for basic, helix-loop-helix, and WRPW domains. Mol Cell Biol 12: 2475–2483. 1588951 10.1128/mcb.12.6.2475-2483.1992 PMC364440

[15] Nolo R, Abbot LA, Bellen HJ (2000) Senseless, a Zn finger transcription factor, is necessary and sufficient for sensory organ development in Drosophila. Cell 102: 349–362. 10975525 10.1016/s0092-8674(00)00040-4

[16] Jarman AP, Sun Y, Jan LY, Jan YN (1995) Role of the proneural gene, *atonal*, in formation of *Drosophila* chordotonal organs and photoreceptors. Development 121: 2019–2030. 7635049 10.1242/dev.121.7.2019

[17] Baker NE, Yu SY (1998) The R8-photoreceptor equivalence group in *Drosophila* : fate choice precedes regulated *Delta* transcription and is independent of *Notch* gene dose. Mechanisms of Development 74: 3–14. 9651468 10.1016/s0925-4773(98)00054-9

[18] Firth LC, Baker NE (2005) Extracellular signals responsible for spatially regulated proliferation in the differentiating Drosophila eye. Dev Cell 8: 541–551. 15809036 10.1016/j.devcel.2005.01.017

[19] Corrigall D, Walther RF, Rodriguez L, Fichelson P, Pichaud F (2007) Hedgehog signaling is a principal inducer of Myosin-II-driven cell ingression in Drosophila epithelia. Dev Cell 13: 730–742. 17981140 10.1016/j.devcel.2007.09.015

[20] Escudero LM, Bischoff M, Freeman M (2007) Myosin II regulates complex cellular arrangement and epithelial architecture in Drosophila. Dev Cell 13: 717–729. 17981139 10.1016/j.devcel.2007.09.002

[21] Bessa J, Gebelein B, Pichaud F, Casares F, Mann RS (2002) Combinatorial control of *Drosophila* eye development by Eyeless, Homothorax, and Teashirt. Genes and Development 16: 2415–2427. 12231630 10.1101/gad.1009002 PMC187435

[22] Firth LC, Baker NE (2009) Retinal determination genes as targets and possible effectors of extracellular signals. Developmental Biology 327: 366–375. 19135045 10.1016/j.ydbio.2008.12.021 PMC2650007

[23] Garrell J, Modolell J (1990) The Drosophila extramacrochaetae locus, an antagonist of proneural genes that, like these genes, encodes a helix-loop-helix protein. Cell 61: 39–48. 1690605 10.1016/0092-8674(90)90213-x

[24] Giagtzoglou N, Alifragis P, Koumbanakis KA, Delidakis C (2003) Two modes of recruitment of E(spl) repressors onto target genes. Development 130: 259–270. 12466194 10.1242/dev.00206

[25] Bai G, Sheng N, Xie Z, Bian W, Yokota Y, et al (2007) Id sustains Hes1 expression to inhibit precocious neurogenesis by releasing negative autoregulation of Hes1. Dev Cell 13: 283–297. 17681138 10.1016/j.devcel.2007.05.014

[26] Alifragis P, Poortinga G, Parkhurst SM, Delidakis C (1997) A network of interacting transcriptional regulators involved in Drosophila neural fate specification revealed by the yeast two-hybrid system. Proc Natl Acad Sci U S A 94: 13099–13104. 9371806 10.1073/pnas.94.24.13099 PMC24269

[27] Baonza A, de Celis JF, Garcia-Bellido A (2000) Relationships between extramacrochaetae and Notch signalling in Drosophila wing development. Development 127: 2383–2393. 10804180 10.1242/dev.127.11.2383

[28] Baker NE, Firth LC (2011) Retinal determination genes function along with cell-cell signals to regulate Drosophila eye development: examples of multi-layered regulation by master regulators. Bioessays 33: 538–546. 21607995 10.1002/bies.201000131 PMC4300108

[29] Li X, Cassidy JJ, Reinke CA, Fischboeck S, Carthew RW (2009) A microRNA imparts robustness against environmental fluctuation during development. Cell 137: 273–282. 19379693 10.1016/j.cell.2009.01.058 PMC2674871

[30] Cronmiller C, Cummings CA (1993) The *daughterless* gene product in *Drosophila* is a nuclear protein that is broadly expressed throughout the organism during development. Mechanisms of Development 42: 159–169. 8217842 10.1016/0925-4773(93)90005-i

[31] Schroeder MD, Pearce M, Fak J, Fan H, Unnerstall U, et al (2004) Transcriptional control in the segmentation gene network of Drosophila. PLoS Biol 2: E271. 15340490 10.1371/journal.pbio.0020271 PMC514885

[32] Zipursky SL, Venkatesh TR, Teplow DB, Benzer S (1984) Neuronal development in the *Drosophila* retina: monoclonal antibodies as molecular probes. Cell 36: 15–26. 6420071 10.1016/0092-8674(84)90069-2

